# On Dyspepsia

**Published:** 1802-05

**Authors:** Robert Kinglake

**Affiliations:** Chilton Super Polden


### On DYSPEPSIA.

To the EDITORS of the MEDICAL and PHYSICAL JOURNAL.

GENTLEMEN,

THE experienced medical practitioner will admit that a large majority of chronic difeafes, and not a fmall proportion of thofe of an acute defcription, owe their origin to indigeftion; nor will it be denied by the moft intelligent of the profeffion, that the difficulties in the way of reftoring the ftomach to a due performance of its digeftive function, are fuch as often to baffle the moft approved modes of treatment. It frequently occurs that the ordinary tribe of ftomachic and tonic medicines, ufually prefcribed in dyfpeptic affections, affords at beft but temporary relief, and that fometimes, by their undue ftimulant influence on the ftomach, they indirectly induce an additional degree of debility on that organ, with the accuftomed train of hurtful confequences, fuch as naufea, anorexia, and fyftematic atony. It has repeatedly happened to me to obferve an irretrievable progrefs in dyfpeptic affections during the protracted and inefficient employ of ftomachic medicines; and on thefe occafions the failure of benefit has apparently arifen more from an implicit reliance on the *sole* efficacy of thefe agents than on the irremediable nature of the affection.

An inveterate diftruft in any thing like uniform medicinal efficiency in the infinitely diverfified nature of organic fufceptibility, firft induced me to try in obftinate cafes of indigeftion the effect of a rigorous attention to a certain plan of domeftic management, in conjunction with the ufual treatment. The advantages refulting from this procedure have enabled me duly to appreciate the importance of the co-operation, and have farther warranted me in expecting that its *solitary* adoption would in general render the conjoint employ of medicine unneceffary in the cure of dyfpeptic diforders.

The treatment alluded to, confifts in cautioufly avoiding mechanical impediments to the procefs of digeftion, and in counteracting that ftomachic languor and irritation which prevent the falutary fecretion of gaftric juice; an agent fo indifpenfably neceffary to the due decompofition and affimilation of alimentary fubftances.

Extenfive experience has now fully confirmed the important fact, that the worft ftates of Dyfpepfia arifing from default in ftomachic excitability, and gaftric fecretion, unaccompanied with with organic læfion, may be effectually remedied by an appropriate regard to the quantity of diet taken at a time, to fuitable maftication, friction over the region of the ftomach, and the avoidance of coftivenefs. The mode of practically applying the conjoint influence of thefe feveral aids, confifts in limiting the quantity of aliment taken at one time to two ounces, in confining it chiefly to animal food, particularly mutton and pork, in mafticating it fo long that it may be reduced by comminution and falival commixture to a femi-fluid ftate. This may be repeated once in three hours. No diluent fluids fhould be taken with the food, nor until one hour after each repaft, left the folvent property of the mixed faliva fhould be thereby diminifhed; nor fhould the quantity of fluid taken at once ever exceed half a pint, nor be repeated oftener than at intervals of three hours.

About half an hour before fwallowing the portion of aliment propofed, brifk friction fhould be performed with a flefh brufh over the region of the ftomach during at leaft ten minutes; a fimilar operation fhould alfo follow it. If the irritability of the fkin over the ftomach fhould difpofe it to inflame, or to throw out eruptions by repeated rubbing, (which fometimes happens) the furface may be fmeared with olive oil previoufly to performing it; on the contrary, fhould neither a glowing fenfe of warmth on the furface, nor any other fenfible impreffion be made by the friction, either powdered muftard-feed, pepper, ginger, or fpirit of wine, may be fprinkled over the fkin before the application of the flefh brufh.

Flatulent diftenfion, and the aching uneafinefs of the ftomach connected with it, are fpeedily fubdued by the ftimulant effect of the friction; and if the precaution be obferved of rubbing circularly from right to left, the redundant gafeous fubftances extricated, and accumulated in the ftomach during indigeftion, will be extruded at the pylorus, or lower orifice of the ftomach, and tranfmitted through the inteftinal canal; while in the contrary, or a more promifcuous direction, they may efcape by the cardia, or upper orifice, and be forced up the œfophagus in fonorous, painful, and unpalatable eructations. An habitual attention to the removal of coftivenefs will powerfully aid the beneficial effects of this treatment. No remedy will be found fo effectual in fubduing this infidious and deftructive enemy to health in general, and to digeftion in particular, as inftituting a regular cuftom of periodically foliciting an evacuation by voluntary and perfevering effort. The morning is the proper feafon for the attempt; it fhould on no occafion be omitted, and the trial fhould be profecuted during at leaft fifteen minutes, if the periftaltic power be not earlier excited to adequate quate motion. A week has been unavailingly employed in this endeavour, but the propofed effect has been attained within a fortnight; and one month has in numerous inftances fully efta-blifhed an *habitual* call to inteftinal evacuation, under circum-ftances that previoufly required the almoft daily ufe of aperient medicine.

Were it neceffary to illuftrate the conjoint efficacy of this treatment by particular cafes, it would be in my power to go into a much more extenfive detail than you may think neceffary to admit in your publication. It may however be proper to remark, that a vaft variety of dyfpeptic affections thus ma-naged, has been recorded by me, many of which had been the caufe, while others had been the effect of chronic maladies, and the whole of them had been more or lefs unfuccefsfully com-bated with the ufual medicinal means of relief. Cafes of a defcription fo deplorably forlorn have been remedied by this management, that it would be much more fatisfactory to me to have a fimilar effect repeated in the experience of others, than by ftating them to rifk incurring any doubt of the credibility of the relation. Nor has the fuccefs alluded to been partial, or felected; a degree of uniformity has indeed marked the falutary influence of the practice but rarely found in the medicinal treat-ment of difeafe.

In fome cafes, the cure has been both perfect and perma-nent; in others, unexpected relief has been afforded; and in all, fufficient benefit has been rendered to encourage an un-wearied profecution of the means.

In reflecting on the mode of remedy here propofed for dyf-peptic affections, it may be allowable to obferve, that the fafhi-onable employ of medicine on all occafions of difeafe, has often led to an unguarded neglect of co-operative aid from domeftic and felf-management; as if the powers of life, and the pecu-liar conditions of health, were not more under the dominion of *ordinary* than *extraordinary* agents; as if the ftomach, for example, were not more likely to return to its regular digeftive function, when folicited by the congenial influence of an ap-proach to its natural ftate, than when difturbed, tortured, and all but diforganized, by the almoft intolerable ftimulant im-preffions of *reputed* ftomachic medicines; and thefe too em-ployed (as if the curative intention were to be accomplifhed by an abfolute *fiat*) under a lax regard, or even total indif-ference to collateral affiftance.

The healing art confifts in inftituting the moft appropriate modes of curing difeafes, not in uniformly wielding againft them medicinal fubftances.

If health can be regenerated by regulations calculated to reproduce its indifpenfable conditions, the falutary end of medicine is happily attained, without the loathfome neceffity of reforting to the employ of noifome drugs.

Without wifhing unduly to curtail the multifarious extent of the materia medica, humanity, it may be allowed, muft triumph in every fair opportunity of fubftituting domeftic for medicinal remedies.

While the dyfpeptic patient will be fatisfied with deriving the defired benefit from the treatment here recommended, the philofophic medical practitioner will readily explain the way in which falutary confequences are likely to refult from its employ. The function of digeftion is perhaps more fcientifically underftood than any other in the animal œconomy. The importance of the gaftric fecretion is admitted, and a due fupply of faliva is alfo known to be effentially requifite in facilitating the changes that are to be effected on alimentary fubftances in the ftomach.

The fupply of the gaftric juice and faliva, in due quantity and quality, will depend on the healthy excitability of the ftomach and falival glands; thus, in impaired digeftion, the attention fhould be chiefly directed to adjufting thefe neceffary conditions.

In directing a limited quantity of food oppreffion is obviated, the motive powers of the ftomach are adequately excited and unclogged, while the elaborate maftication recommended, and the confequently copious commixture of faliva, leave lefs to be accomplifhed by the decompofing influence of the gaftric juice.

Friction on the abdomen excites motion, which, by affociated efficiency, is propagated to the ftomach, and ferves to impart additional activity to the digeftive procefs.

Neither fimilarity of ftructure, nor direct continuity of connection, fubfifts between the cuticular furface over the region of the ftomach, and the fubftance of that vifcus, yet an intimate confent of motive power obtains between thefe feveral parts, which explains how topical friction extends falutary influence to the organ itfelf.

It is a fuccedaneum for corporeal exercife, and by being concentrated on the ftomach, proves a more direct remedy in the removal of flatulency, and other morbid inconveniences arifing from indigeftion, than could be afforded by a more general exertion of the fyftem.

The fertile caufes of Dyfpepfia are repletion and inaction. The combined tendency of thefe evils is to induce both ftomachic and fyftematic oppreffion. The neceffary demand for nutriment is varied by the degree of voluntary exertion, fuperadded to the involuntary motions of life; if the former be too limited limited, morbid repletion, both locally and generally, can only be avoided by a moderate ufe of diet; on the contrary, if the habitual exercife be carried the length of inducing frequent perfpiration, and mufcular fatigue, the exigencies for alimentary fupport will be proportionately great; hence an exact ratio fubfifts between the exhauftion from exercife and the want of nutritive fupply. If this balance, fo accurately ftruck by Nature, were uniformly fuftained in the conduct of mankind, Dyfpepfia, and the hydra train of mifchief it is liable to produce, would fcarcely be known in the catalogue of human afflictions.

The plan of remedying indigeftion here recommended on the authority of abundant experience, is further warranted by the analogy it bears to the agency ufually obtaining in the procefs of natural digeftion. In the one cafe, ftomachic oppreffion is obviated by fmall portions of food taken at a time, by affiduous maftication, falival commixture, and inteftinal regularity, while digeftion is much promoted by abdominal friction; in the other, or the ordinary performance of this function, perfonal exercife becomes a fubftitute for thefe precautions; it accelerates the animo-chemical combinations, and decompofitions, which conftitute the motive evolutions of life; and thus requires fuch an extent of nutritive fupply from the digeftive function of the ftomach, as at once obviates morbid plenitude, and induces falutary hunger.

I am, &c.
ROBERT KINGLAKE.

*Chilton Super Polden,*

